# Intracellular ROS Scavenging Activity and Downregulation of Inflammatory Mediators in RAW264.7 Macrophage by Fresh Leaf Extracts of *Pseuderanthemum palatiferum*

**DOI:** 10.1155/2014/309095

**Published:** 2014-03-16

**Authors:** Patcharawan Sittisart, Benjamart Chitsomboon

**Affiliations:** ^1^School of Biology, Institute of Science, Suranaree University of Technology, Nakhon Ratchasima 30000, Thailand; ^2^School of Pharmacology, Institute of Science, Suranaree University of Technology, Nakhon Ratchasima 30000, Thailand

## Abstract

Beneficial antioxidant phytochemicals are found in many medicinal plants. *Pseuderanthemum palatiferum* (PP), a well-known Vietnamese traditional medicinal plant in Thailand, has long been used in folk medicine for curing inflammatory diseases, often with limited support of scientific research. Therefore, this study aimed to determine antioxidant and modulation of inflammatory mediators of ethanol and water extracts of PP (EEP and WEP, resp.). WEP had significantly higher phenolic and flavonoid levels and DPPH radical scavenging activity than EEP. However, EEP exhibited greater reducing power than WEP. A greater decrease of *tert*-butyl hydroperoxide-induced oxidative stress in RAW264.7 macrophage cells was also observed with EEP. Modulation of inflammatory mediators of EEP and WEP was evaluated on LPS plus IFN-**γ**-stimulated RAW264.7 cells. EEP more potently suppressed LPS plus IFN-**γ**-induced nitric oxide (NO) production than WEP. Both EEP and WEP also suppressed the expression of iNOS and COX-2 protein levels. Collectively, these results suggest that PP possesses strong antioxidant and anti-inflammatory properties.

## 1. Introduction

Oxidative stress is known to cause cellular damage linked to various degenerative processes and diseases, such as aging, ischemic injury, atherosclerosis, cancer, diabetes, and various inflammatory diseases [1]. Macrophages are key players in inflammation and their activation is crucial in inflammatory processes [2]. Many inflammatory stimuli including bacterial lipopolysaccharide (LPS) and IFN-*γ* can stimulate macrophages to produce proinflammatory cytokines and small mediators, such as nitric oxide (NO) and prostaglandin E2 (PGE_2_) [3]. Excess levels of NO produced by activated macrophages reflect the inflammation process and are regulated by inducible nitric oxide synthase (iNOS) [4]. Overproduction of NO has been known to be associated with various diseases, such as cancer, rheumatoid arthritis, septic shock, autoimmune disease, and chronic inflammation [5]. PGE_2_, the key player in inflammatory response, is produced from arachidonic acid by prostaglandin synthase or cyclooxygenase (COX) enzymes. COX exists as two isoforms: COX-1 and COX-2. COX-1 is constitutively expressed and is a housekeeping enzyme required for normal physiological functions. COX-2 is considered as the inducible isoform and is primarily involved in inflammation [6]. Linkage and cross talk among NO, iNOS, and COX-2 during the inflammation process are well established. NO directly enhances COX-2 activity which results in a remarkable synthesis of PGE_2_. iNOS and COX-2 can work together in a variety of similar pathophysiological actions and inflammatory diseases [5, 7]. In addition, many inflammatory effects have been reported to be associated with high productions of NO, iNOS, and COX-2 [8]. Therefore, an agent with inhibitory effects on excess levels of NO, iNOS, and COX-2 expression would be highly beneficial and part of an effective strategy in the treatment of inflammatory diseases.

Over the last decade medicinal plants as potential sources of naturally occurring antioxidants have been the focus of intense research. Moreover, phytochemicals such as flavonoids and other polyphenolics with high reactive oxygen species (ROS) scavenging activities have been shown to exhibit multiple biological effects, including antiallergic, antibacterial, antidiabetic, anticancer, and anti-inflammatory activities [9]. As oxidative stress and inflammation are closely linked and are implicated in many diseases [10], plants that possess both antioxidant and anti-inflammatory properties have always attracted considerable research interest.* Pseuderanthemum palatiferum *(Nees) Radlk. (PP), a member of the Acanthaceae plant family and commonly called Hoan-Ngoc, is one of the most popular medicinal plants in both Thailand and Vietnam. Phytochemical analysis of PP leaf extracts suggests many high potential antioxidant and anti-inflammatory constituents [11]. In fact, PP has been referred to as a miracle plant in folk medicine to cure or prevent various maladies and inflammatory related diseases such as diarrhea, sore throat, hypertension, gastric ulcer, diabetes, and cancer [12, 13]. Nevertheless, the scientific evidence to support its multiple biological effects is still limited, particularly related to anti-inflammation. Although the antioxidant property of PP leaf extracts has been previously shown [14, 15], its intracellular ROS scavenging activity has never been assessed. To date, there is only one study reporting the anti-inflammatory activity of PP leaf extract [13], and the mechanism responsible for the anti-inflammation remains largely unknown. This study further compares antioxidant activity between ethanol and water extracts of PP leaves using various* in vitro* antioxidant evaluation methods including the assessment in the cell-based DCFH-DA assay. The modulation of PP leaf extracts in NO production, iNOS, and COX-2 expression during the inflammatory response was also investigated in the murine macrophage-like cell line RAW264.7 stimulated with LPS plus IFN-*γ*.

## 2. Materials and Methods

### 2.1. Chemicals and Materials

3-(4,5-Dimethylthiazol-2-yl)-2,5-diphenyltetrazolium bromide (MTT), (+)-catechin hydrate, and vitamin C were purchased from Fluka Chemie GmbH (Buchs, Switzerland). 2,2-Diphenyl-1-picryl-hydrazyl (DPPH), penicillin G, streptomycin sulfate, resveratrol, N-(1-naphthyl)ethylenediamine dihydrochloride (NED), sodium nitrite, LPS (*Escherichia coli* O111:B4), 2′,7′-dichlorofluorescin-diacetate (DCFH-DA), and* tert*-butyl hydroperoxide (tBuOOH) were purchased from Sigma-Aldrich (St. Louis, MO, USA). Dimethyl sulfoxide (DMSO) was purchased from Amresco Inc. (Solon, OH, USA). Quercetin dihydrate was obtained from INDOFINE Chemical Company, Inc. (Hillsborough, NJ, USA). 6-Hydroxy-2,5,7,8-tetramethylchroman-2-carboxylic  acid (Trolox) was purchased from Sigma-Aldrich Chemie GmbH (Steinheim, Germany). Mouse interferon gamma (mIFN-*γ*) and ECL Western blotting substrate were purchased from Pierce Protein Research Products (Rockford, IL, USA). RPMI medium 1640, Hank's balanced salt solution (HBSS), and penicillin-streptomycin were obtained from Gibco Invitrogen (Grand Island, NY, USA). Fetal bovine serum (FBS) was obtained from Hyclone (Logan, UT, USA). Anti-iNOS and anti-tubulin mouse monoclonal antibodies and secondary antibody goat-anti-mouse-HRP conjugate for iNOS and tubulin were purchased from Santa Cruz Biotechnology Inc. (Santa Cruz, CA, USA). Anti-COX-2 mouse polyclonal antibody and secondary antibody goat-anti-rabbit IgG-HRP conjugate for COX-2 were purchased from Cayman Chemical (Ann Arbor, MI, USA). The mouse macrophage cell line (RAW264.7 cells) was purchased from Cell Lines Service (Eppelheim, Germany). All other reagents were purchased from Sigma-Aldrich unless otherwise indicated.

### 2.2. Plant Material

Fresh leaves of PP were purchased from producers in Yasothon province, Thailand. The plant was identified and authenticated by Dr. Kongkanda Chayamarit, Forest Herbarium, Royal Forest Department, Bangkok, Thailand. A voucher specimen (BKF 174009) was deposited at the Forest Herbarium, Royal Forest Department, Bangkok, Thailand.

### 2.3. Plant Extract Preparation

Fresh leaves (1.5 kg) were cut into small pieces and blended in 6 L of 95% ethanol. The extract was centrifuged at 3,500 g for 10 min at 4°C and the supernatant was filtered through Whatman number 1 filter paper. The ethanolic filtrate was then concentrated using a vacuum rotary evaporator and lyophilized to obtain the ethanol extract of PP (EEP; 60.41 g). Forty grams of EEP were further partitioned between hexane and water (1 : 1) using a separatory funnel. The water fraction was collected, centrifuged at 14,000 g for 10 min at 4°C, evaporated, and lyophilized to obtain a water extract of PP (WEP; 32.71 g). The EEP and WEP were stored at −20°C until they were needed in subsequent experiments. The EEP and WEP were dissolved in DMSO and water, respectively, when used in experiments. For cell cultures, the WEP was dissolved in phosphate buffered saline (PBS).

### 2.4. Total Phenolic Content

The total phenolic content of the individual extract was determined by the method of Folin-Ciocalteu [16]. Briefly, 100 *μ*L of test solution was added to 2 mL of 2% Na_2_CO_3_ and mixed thoroughly. After 2 min, 100 *μ*L of 50% Folin-Ciocalteu reagent was added, mixed, and allowed to stand at room temperature (RT) for 30 min. The absorbance of extracts was measured at 750 nm by a Cecil 1000 series spectrophotometer (Cecil Instruments, Cambridge, UK) against a blank consisting of only reagents and solvents without the extract. Gallic acid solutions ranging from 0.05 to 0.3 mg/mL were used to prepare a standard curve. The concentration of phenolic compounds in the extracts is expressed as mg of gallic acid equivalent (GAE) per g of dry extract.

### 2.5. Total Flavonoid Content

The total flavonoid content was determined using a colorimetric method [17]. Briefly, 250 *μ*L of sample was diluted with 1.25 mL of distilled water (DI). Then 75 *μ*L of 5% NaNO_2_ solution was added to the mixture. After 6 min, 150 *μ*L of a 10% AlCl_3_·6H_2_O solution was added, and the mixture was allowed to stand for another 5 min. One half mL of 1 M NaOH was added, and the total volume was brought up to 2.5 mL with DI water. The solution was thoroughly mixed, and the absorbance was measured immediately against the prepared blank at 510 nm. Catechin standard solutions (0.05–0.4 mg/mL) were used to prepare a standard curve. The concentration of flavonoids in the extracts is expressed as mg of catechin equivalent (CAE) per g of dry extract.

### 2.6. FRAP (Ferric Reducing Antioxidant Power) Assay

The ferric reducing ability of the extracts was measured colorimetrically according to the method developed by Benzie and Strain [18]. The FRAP reagent consisted of 0.1 M acetate buffer (pH 3.6), 10 mM 2,4,6-tris(2-pyridyl)-1,3,5-triazine (TPTZ) solution in 40 mM HCl, and 20 mM FeCl_3_·6H_2_O solution. The fresh working solution was prepared by mixing the acetate buffer, the TPTZ solution, and the FeCl_3_·6H_2_O solution in a 10 : 1 : 1, v/v/v, ratio. The FRAP reagent (3 mL) was added to 0.1 mL of the extract and mixed. Readings were recorded on the spectrophotometer at 593 nm, and the reaction was monitored for 10 min. A standard curve of 100–1,000 *μ*mol FeSO_4_·7H_2_O was prepared. Vitamin C (10–90 *μ*g/mL), Trolox (10–160 *μ*g/mL), and catechin (10–90 *μ*g/mL) were used as standard antioxidants. The antioxidant power of the extracts is expressed as mmol ferrous ion (Fe^2+^) per g of dry extract and also mg of vitamin C equivalent (VCE), Trolox equivalent (TRE), and catechin equivalent (CAE) per g of dry extract.

### 2.7. DPPH Assay

The scavenging activity of DPPH radicals was determined as described by Sánchez-Moreno et al. [19]. Briefly, 100 *μ*L of extract at different concentrations was added to 3.9 mL of methanolic DPPH solution (63 mM). The mixture was shaken vigorously and left to stand at RT for 45 min in the dark. Samples that are able to scavenge DPPH free radicals reduce the purple DPPH radicals into the light yellow colored product of corresponding hydrazine DPPH_2_. Decreasing DPPH solution absorption (measured spectrophotometrically at 515 nm) indicates an increase of DPPH radical scavenging activity [20]. DPPH solution plus methanol were used as negative control, and vitamin C, Trolox, and catechin were used as positive controls. The percent inhibition of DPPH radicals by test samples was determined by comparison with the methanol-treated control. The free radical scavenging activity which is the percentage inhibition of free radical is calculated as follows:

(1) DPPH radical scavenging activity (%)    =[Acontrol−AsampleAcontrol]×100,

where *A*_sample_ and *A*_control_ are absorbances of the sample and the control, respectively. The IC_50_ of DPPH radicals was determined from a dose response of inhibitory curve using linear regression analysis.

### 2.8. Cell Culture

The RAW264.7 macrophage cells were cultured at 37°C, 5% CO_2_ in an RPMI-1640 medium supplemented with 10% heat-inactivated FBS, 100 U/mL penicillin, and 100 *μ*g/mL streptomycin. Exponentially growing cells were used for experiments when they reached about 80% confluence.

### 2.9. Cell Viability (MTT Assay)

A tetrazolium dye (MTT) colorimetric assay was used to determine the viability of RAW264.7 cells as described by Chun et al. [21]. Briefly, RAW 264.7 cells were plated at a density of 5 × 10^4^ cells/well in a 96-well plate and incubated overnight at 37°C under 5% CO_2_. After incubation, the cells were exposed to various concentrations of EEP or WEP for 24 h. Then, MTT (0.5 mg/mL) dye solution was added in each well and further incubated at 37°C, 5% CO_2_ for 4 h. The media was removed and DMSO was added to each well to dissolve formazan crystals giving a uniform dark purple color before reading at 540 nm by the Benchmark Plus Microplate Spectrophotometer System (Bio-Rad Laboratories, Inc., Hercules, CA, USA). The percentage of cell viability was calculated by the following equation:

(2) $$\begin{array}{c} \text{Percent cell viability}=\frac{{\text{OD}}_{\text{test group}}}{{\text{OD}}_{\text{control group}}}\times 100. \end{array}$$

### 2.10. Assessment of Intracellular ROS Scavenging Activity

Intracellular oxidative stress was detected using DCFH-DA as described by Kim et al. [22] with slight modification. Briefly, RAW264.7 cells (4 × 10^4^ cells/well) were plated in a Costa 96-well black clear bottom plate (Corning Inc., Corning, NY, USA) and incubated for 16–18 h at 37°C and 5% CO_2_. After incubation, the cells were washed with PBS twice. To assess antioxidant activity, the cells were preexposed to different concentrations of EEP, WEP (50, 150, or 250 *μ*g/mL) or the antioxidant positive controls, catechin (250 *μ*M), resveratrol (20 *μ*M), or quercetin (10 *μ*M), for 24 h. After washing twice with PBS, the cells were exposed to 20 *μ*M DCFH-DA in HBSS and further incubated in the dark for another 30 min. The DCFH-DA was removed by washing the cells with PBS two times. Then, 500 *μ*M tBuOOH was added. The unstimulated DCFH-DA (no tBuOOH) in the unexposed RAW264.7 cells served as the naive control (NA). The intensity of the fluorescence signal was detected time dependently with an excitation wavelength of 485 nm and an emission wavelength of 535 nm using a Gemini EM fluorescence microplate reader (Molecular Devices, Sunnyvale, CA, USA).

### 2.11. Nitrite Assay

The level of NO in the culture media was detected as nitrite, a major stable product of NO, using Griess reagent [23]. RAW264.7 cells were seeded at a density of 2 × 10^5^ cells/well in a 96-well plate. The cells were grown for 3 h to allow plate attachment prior treating with the antioxidant positive control vitamin C (500 *μ*M) or various concentrations (50, 100, 150, 200, or 250 *μ*g/mL) of EEP or WEP. After 1 h incubation, the RAW264.7 cells were stimulated with 1 *μ*g/mL LPS plus 25 U/mL IFN-*γ*. The activated cells were further incubated for 24 h. Then, 100 *μ*L of supernatant was mixed with an equal volume of Griess reagent (1% sulfanilamide, 0.1% NED, and 3% phosphoric acid). After 10 min of incubation in the dark, the absorbance of samples was measured at 540 nm using a Microplate Spectrophotometer System (Bio-Rad Laboratories, Inc.). A fresh culture medium was used as the blank in all experiments. The amount of nitrite in the samples was derived from a standard curve of sodium nitrite.

### 2.12. Western Blot Analysis

RAW264.7 cells were plated at a density of 2 × 10^6^ cells/well in a 6-well plate. After an attachment period of approximately 3 h, the cells were treated with various concentrations (50, 100, 150, 200, or 250 *μ*g/mL) of EEP or WEP for 1 h. 50 *μ*g/mL Trolox or 500 *μ*M vitamin C was used as antioxidant positive controls. The cells were then stimulated with 1 *μ*g/mL LPS plus 25 U/mL IFN-*γ* for 18 h. After incubation, the cells were washed three times with PBS and placed in 150 *μ*L of ice-cold lysis buffer (1 mL RIPA buffer supplemented with 2 mM PMSF, 2 *μ*M leupeptin, and 1 *μ*M E-64) for 20 min. Then the disrupted cells were transferred to microcentrifuge tubes and centrifuged at 14,000 g at 4°C for 30 min. The supernatant was collected and the protein concentration of cell lysate was estimated by the Lowry method [24]. Cell lysate was then boiled for 5 min in a 6X sample buffer (50 mM Tris-base, pH 7.4, 4% SDS, 10% glycerol, 4% 2-mercaptoethanol, and 0.05 mg/mL of bromophenol blue). Thirty micrograms of cellular proteins were separated by sodium dodecyl sulfate-polyacrylamide gel electrophoresis (SDS-PAGE) using 7.5% and 10% polyacrylamide gels for iNOS and COX-2, respectively (125 volts, 120 min). The proteins in the gel were transferred onto a nitrocellulose membrane (Amersham, Pittsburgh, PA, USA) at 80 volts for 1 h. The membrane was blocked overnight at 4°C with 5% nonfat milk in 0.1% Tween 20 in a PBS buffer (TPBS). The membranes were then incubated with a 1 : 1,000 dilution of the primary antibody anti-iNOS mouse monoclonal or a 1 : 2,000 dilution of the primary antibody anti-COX-2 mouse polyclonal at RT for 2 h. After extensive washing with TPBS, the membranes were incubated with a 1 : 10,000 dilution of the secondary antibody goat-anti-mouse-HRP conjugate for iNOS and goat-anti-rabbit IgG-HRP conjugate for COX-2 at RT for 1 h. To control equal loading of total protein in all lanes, blots were also stained with primary antibody anti-tubulin mouse monoclonal at a dilution of 1 : 2,000 at RT for 2 h. After washing, the membranes were incubated with a 1 : 10,000 dilution of the secondary antibody goat-anti-mouse-HRP conjugate. The membranes were washed three times, for 10 min each time, with TPBS. The blots were incubated for 3 min in ECL Western blotting substrate and exposed to film. The relative expression of proteins was quantified densitometrically using the software imageJ and calculated according to the reference band of tubulin.

### 2.13. Statistical Analysis

All statistical analyses were conducted using GraphPad software (GraphPad Prism 5, USA). The data from the total phenolic and flavonoid contents as well as FRAP value results were analyzed by a Student's *t*-test to determine the statistical significance between two groups. DPPH, MTT, and nitrite assays were analyzed by one-way analysis of variance (ANOVA) with a* post hoc* Tukey's analysis to determine differences between treatment and control groups [25]. The data from intracellular ROS scavenging were analyzed by two-way ANOVA followed by Bonfferonni's* post hoc *test [26].

## 3. Results

### 3.1. The Percentage of Recovery of Crude Extracts from Fresh Leaves of PP

The percentages of recovery of crude extracts from fresh leaves of PP are shown in Table 1. EEP exhibited a percentage of recovery of 4.03%, while WEP had percentage of recovery of 3.29% based on the original weight of fresh leaves. WEP was prepared from the water fraction of EEP that was partitioned with hexane and water (1 : 1, v/v) with a percentage of recovery of 81.77% based on EEP.

### 3.2. Phenolic and Flavonoid Contents

WEP had a significantly higher level (*P* < 0.05) of total phenolic and flavonoid content than that of EEP (Table 2), and more than half of the phenolics in WEP and EEP are flavonoids.

### 3.3. Ferric Reducing Antioxidant Power

EEP and WEP were analyzed for their reducing ability along with three standard antioxidants, vitamin C, Trolox, and catechin. The results of FRAP values in terms of ferrous ion (Fe^2+^) and vitamin C, Trolox, and catechin equivalents are shown in Table 2. EEP exhibited a higher degree of electron donating capacity than WEP as suggested by the significantly higher FRAP values (*P* < 0.05) of EEP when compared with WEP.

### 3.4. DPPH Free Radical Scavenging Activity

**Figure 1:**
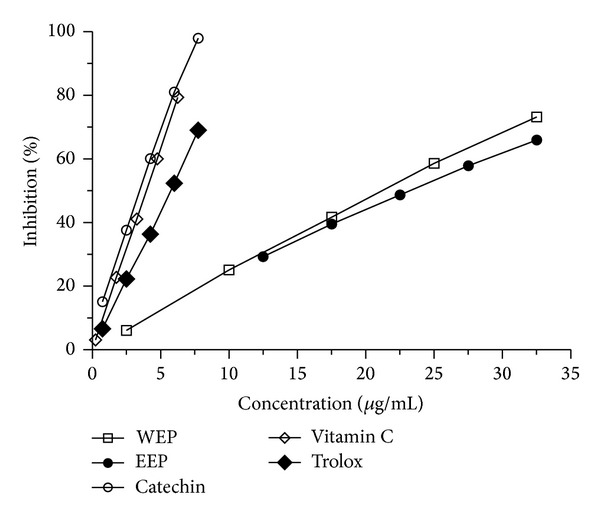
DPPH radical scavenging activity of PP leaf extracts (EEP and WEP) and positive controls (vitamin C, Trolox, and catechin). Values are means ± SEM (*n* = 3) and are representative of three independent experiments with similar results.

The free radical scavenging capacities of EEP and WEP are shown in Figure 1. The results show that both EEP and WEP exhibit the ability to scavenge DPPH free radicals. The scavenging activity against DPPH radicals of WEP (IC_50_ = 21.55 ± 0.06 *μ*g/mL) is significantly greater (*P* < 0.001) than EEP (IC_50_ = 23.45 ± 0.12 *μ*g/mL) by 1.9 ± 0.15%, but the scavenging capacity of these is not as effective as the other positive antioxidant controls. 12.5 *μ*g/mL EEP and 2.5 *μ*g/mL WEP scavenged the DPPH radicals by 29.27 ± 0.20% and 6.12 ± 0.15%, respectively, and the scavenging capacities of both are more pronounced at higher concentrations. The highest concentration (32.5 *μ*g/mL) of EEP and WEP could scavenge the DPPH radicals by 65.96 ± 0.21% and 73.19 ± 0.09%, respectively. In the present study, the scavenging abilities of vitamin C (IC_50_ = 3.94 ± 0.01 *μ*g/mL) and catechin (IC_50_ = 3.55 ± 0.01 *μ*g/mL) were similar, and both are significantly higher (*P* < 0.001) than Trolox (IC_50_ = 5.90 ± 0.27 *μ*g/mL).

### 3.5. Effect of EEP and WEP on RAW264.7 Cell Viability

**Figure 2:**
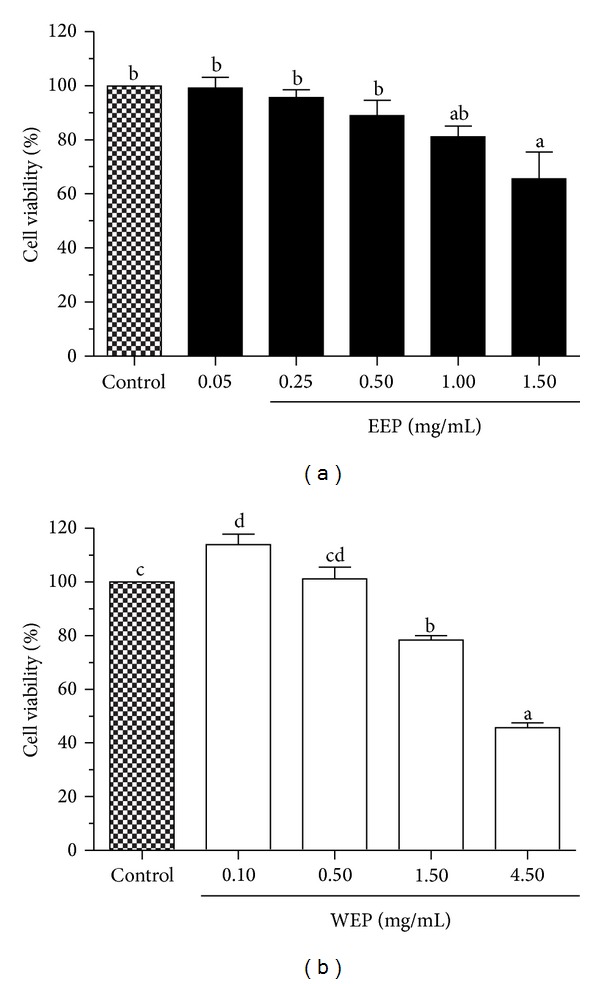
Effect of EEP and WEP on cell viability of RAW264.7 cells. The effect of EEP (a) and WEP (b) on cell viability was assessed by MTT. Values are expressed as means ± SEM (*n* = 3) and are representative of three independent experiments with similar results. Bars marked with different letters are significantly different at *P* < 0.05 as determined by one-way ANOVA.

The cell viability of RAW264.7 cells exposed to EEP or WEP was determined by MTT assay. The cells were incubated for 24 h with various concentrations of EEP (0.05, 0.25, 0.5, 1.0, or 1.50 mg/mL) or WEP (0.10, 0.50, 1.50, or 4.50 mg/mL). As shown in Figure 2, both EEP and WEP displayed low toxicity towards RAW264.7 cells as evidenced by an apparent lack of effect on cell viability until the concentration of each extract reached 1.5 mg/mL. At 1.5 mg/mL, EEP and WEP decreased the viability of RAW264.7 cells by 34.14 ± 9.69% and 21.58 ± 1.66% (*P* < 0.05), respectively. However, the cytotoxic effect is more pronounced at higher concentrations. WEP at 4.5 mg/mL decreased the cell viability by as much as 54.21 ± 1.74% (*P* < 0.05). The effect of EEP and WEP on RAW264.7 cell viability was also confirmed by trypan blue exclusion and propidium iodide staining methods, which exhibited similar results (data not shown). Therefore, a nontoxic concentration range of 0–0.25 mg/mL of both EEP and WEP was selected for RAW264.7 cell treatment in the subsequent studies.

### 3.6. EEP and WEP as Intracellular ROS Scavengers

**Figure 3:**
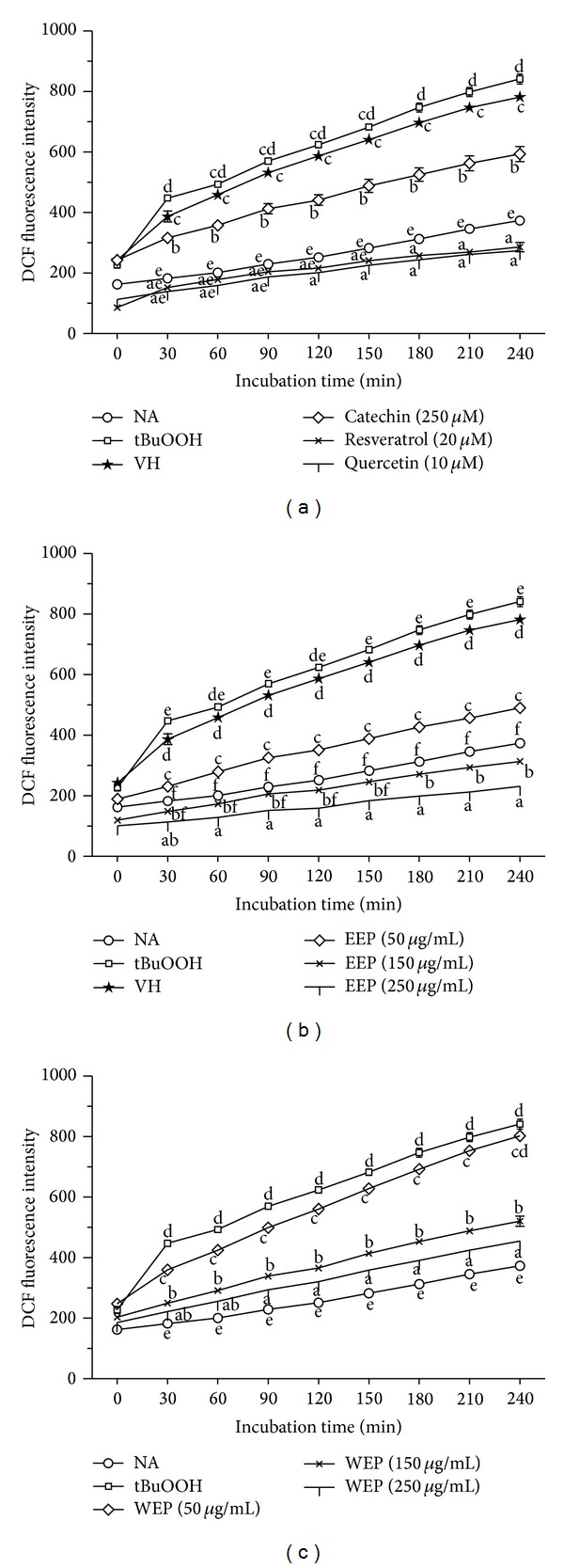
Cellular radical scavenging activity in tBuOOH-activated RAW264.7 cells. Intracellular ROS level generated in cells was measured by the DCFH-DA. RAW264.7 cells were pretreated with indicated concentrations of antioxidants (a), EEP (b) or WEP (c), for 24 h prior to take-up of 20 *μ*M DCFH-DA for 30 min. Results are mean ± SEM (*n* = 4) and are representative of three independent experiments with similar results. Points marked with different letters are significantly different at *P* < 0.05 when compared at the same time point as determined by two-way ANOVA.

The direct scavenging effect of EEP and WEP on intracellular free radical stress was investigated in RAW264.7 cells using the DCFH-DA assay. The increment of DCF fluorescence emission following ROS-mediated oxidation of DCFH was followed for 240 min. As shown in Figure 3(a), standard antioxidant positive controls, catechin (250 *μ*M), resveratrol (20 *μ*M), and quercetin (10 *μ*M), could scavenge ROS significantly (*P* < 0.05) throughout the incubation time when compared to the vehicle control (VH). With as little as 30 min of incubation, catechin, resveratrol, and quercetin showed considerable radical scavenging activity. EEP (Figure 3(b)) and WEP (Figure 3(c)) decreased the DCF fluorescent emission in a dose- and time-dependent manner. Again, with as little as 30 min of incubation, both EEP and WEP at low concentration (50 *μ*g/mL) showed similar radical scavenging activity as the antioxidant controls. Various concentrations of EEP significantly decreased (*P* < 0.05) the DCF fluorescent emission throughout the incubation time when compared to the VH control. At high concentration (150 *μ*g/mL), EEP exhibited a strong scavenging activity as suggested by the capability to lower fluorescent intensity to below basal level of the unstimulated DCFH-DA control at 180–240 min. In addition, the highest concentration of EEP (250 *μ*g/mL) significantly lowered (*P* < 0.05) DCF fluorescent intensity to below the basal level at all time points. Similarly, 150 and 250 µg/mL of WEP also significantly decreased (*P* < 0.05) the DCF fluorescent emission throughout the incubation time compared to the tBuOOH control. However, the lowest concentration of WEP (50 *μ*g/mL) significantly reduced (*P* < 0.05) the DCF fluorescent emission until 210 min only.

### 3.7. NO Suppression by EEP and WEP in LPS Plus IFN-*γ*-Activated RAW264.7 Cells

**Figure 4:**
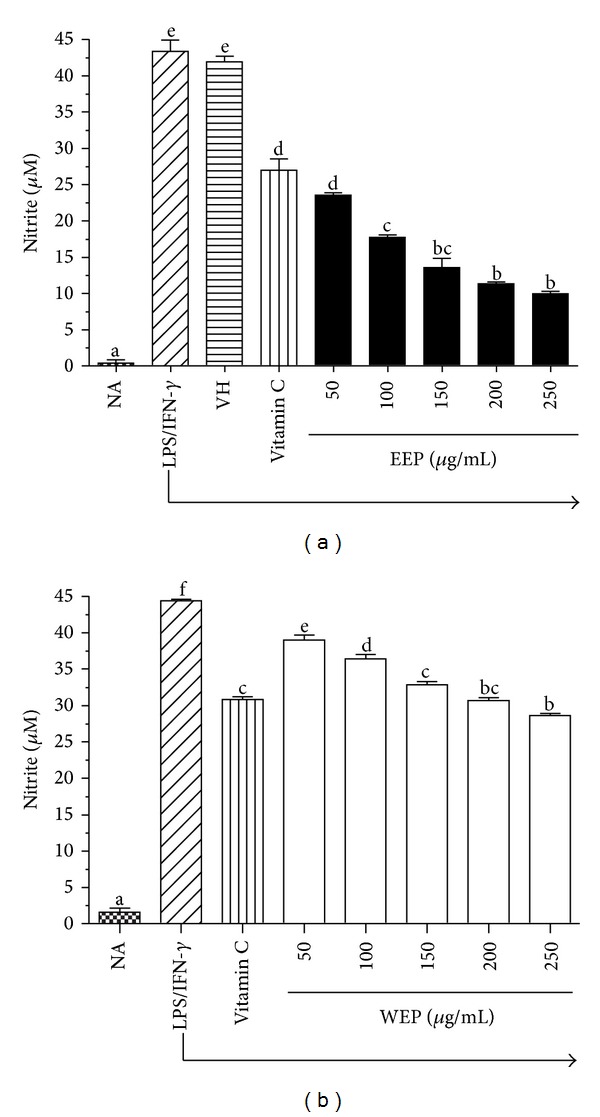
EEP (a) and WEP (b) suppressed LPS plus IFN-*γ*-induced nitrite production in RAW264.7 cells. RAW264.7 cells were incubated for 24 h with LPS (1 *μ*g/mL) plus IFN-*γ* (25 U/mL) in the presence or absence of indicated concentrations of vitamin C (500 µM), EEP, or WEP. Accumulated nitrite in the culture medium was determined by the Griess reaction. The values are means ± SEM (*n* = 3) and are representative of three independent experiments with similar results. Bars marked with different letters are significantly different at *P* < 0.05 as determined by one-way ANOVA.

RAW264.7 cells were pretreated with antioxidants, vitamin C, EEP, or WEP for 1 h, then stimulated with LPS plus IFN-*γ*, and measured for NO production using the Griess assay. As shown in Figure 4, unstimulated RAW264.7 cells (NA) secreted basal levels of NO, while the production of NO was increased to about 43 *μ*M in LPS plus IFN-*γ*-activated RAW264.7 cells. The antioxidant control, 500 *μ*M vitamin C, decreased the NO production by almost 35%. Pretreatment of RAW264.7 cells with EEP or WEP significantly suppressed (*P* < 0.05) the induction of NO in a dose-related manner (Figures 4(a) and 4(b)), and the suppression was observed in all EEP- and WEP-treated groups. These results also clearly indicate that EEP is a stronger suppressant of NO induction than WEP. Concentrations of 50 *μ*g/mL of EEP and 150 and 200 *μ*g/mL of WEP were required to exhibit the NO suppression with the same efficiency as 500 *μ*M (88.06 *μ*g/mL) vitamin C.

### 3.8. Suppression of iNOS and COX-2 Protein Expression by EEP and WEP in LPS Plus IFN-*γ*-Activated RAW264.7 Cells

**Figure 5:**
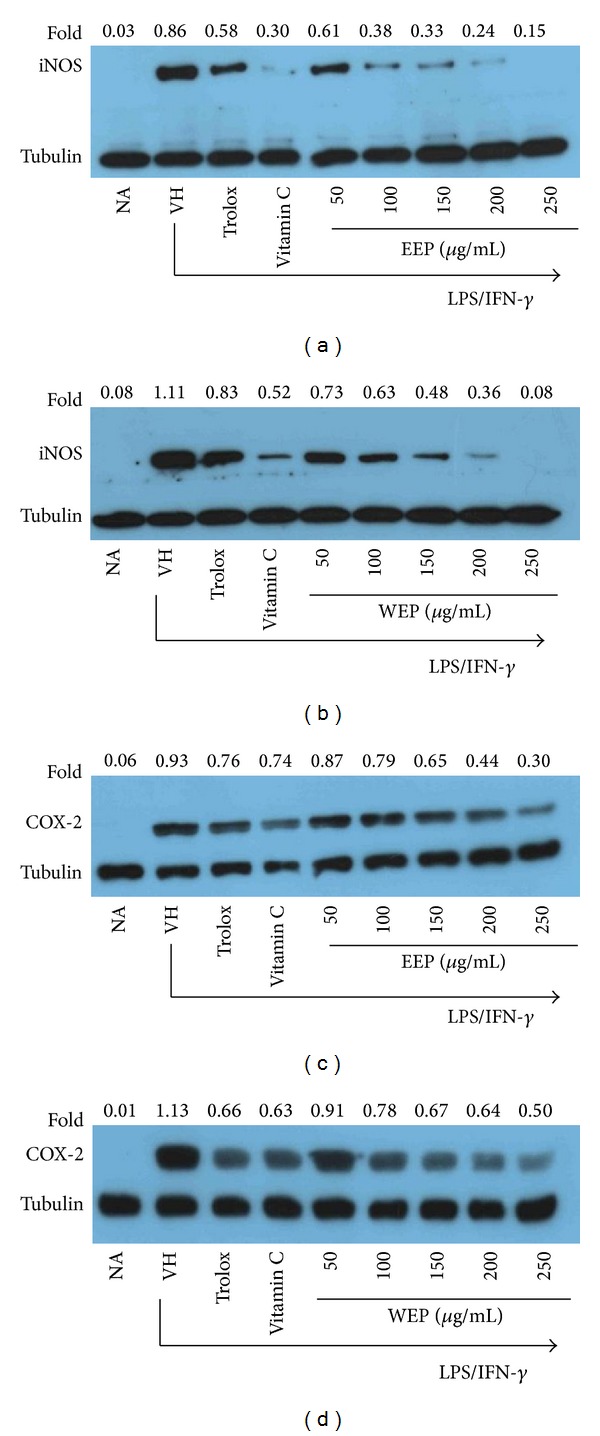
Effect of EEP on LPS plus IFN-*γ*-induced iNOS (a) and COX-2 (c) and WEP on LPS plus IFN-*γ*-induced iNOS (b) and COX-2 (d) protein levels in RAW264.7 cells. The relative expression of proteins was quantified densitometrically using ImageJ software and normalized to tubulin reference bands. Data are representative of at least two independent experiments.

To determine if suppression of NO production by EEP or WEP was related to changes in iNOS as well as COX-2 protein levels, Western blotting analysis was performed. RAW264.7 cells were pretreated with antioxidants, Trolox (50 *μ*g/mL), vitamin C (500 *μ*M), or PP extracts (EEP or WEP) at 50–250 µg/mL for 1 h prior activation with LPS (1 *μ*g/mL) plus IFN-*γ* (25 U/mL) for 18 h. Total proteins were extracted and analyzed for the expression of iNOS and COX-2 by Western blotting. LPS plus IFN-*γ* induced increases in iNOS (Figures 5(a) and 5(b)) and COX-2 (Figures 5(c) and 5(d)) expression compared to the unstimulated cultures. Antioxidant controls (Trolox and vitamin C) decreased LPS plus IFN-*γ*-induced iNOS and COX-2 protein levels. The data also suggested that the suppression by 500 *μ*M (88.06 *μ*g/mL) vitamin C is more pronounced than 50 *μ*g/mL Trolox. Compared to the corresponding controls, both EEP and WEP produced a dose-dependent suppression of iNOS level in LPS plus IFN-*γ*-activated RAW264.7 cells (Figures 5(a) and 5(b)), suggesting that the suppression of NO production by EEP and WEP is mediated by decreasing the expression of iNOS. In agreement with the result of NO suppression, 50–200 *μ*g/mL EEP was probably more efficient than WEP in iNOS suppression. The iNOS expression was almost completely eliminated at 200 *μ*g/mL EEP and was barely observed at 250 µg/mL WEP. The inflammatory modulation of EEP and WEP was also further supported by the dose-dependent suppression of the COX-2 level by both EEP and WEP (Figures 5(c) and 5(d)) in the activated RAW264.7 cells. Notably, EEP and WEP exhibited higher suppression of iNOS than COX-2.

## 4. Discussion

It is well known that major phytochemicals of plant leaf extracts possessing antioxidant activity are flavonoids and other phenolic compounds. Researchers have found that flavonoids from PP leaves display antioxidant activity and all ethyl acetate, chloroform, and *n*-butanol-soluble fractions of PP contain flavonoids [11, 15]. In addition, Nguyen and Eun [14] found phenolics and flavonoids in extracts of PP leaves when assessed with Folin-Ciocalteu and aluminum trichloride. PP leaf extracts also have antioxidant activities when evaluated with DPPH and FRAP assays. Similarly, the present study also showed that both EEP and WEP contain high levels of flavonoids and phenolics and exhibit antioxidant activity. The most frequently used antioxidant standards for food samples (vitamin C, Trolox, catechin, resveratrol, and quercetin) were used as positive antioxidant controls in the present study.

This study revealed that DPPH radical scavenging capacity of WEP is greater than that of EEP (Figure 1). In contrast, EEP has higher ferric reducing power than WEP (Table 2). Such contradictory results between DPPH and FRAP assays are not unusual. Though both assays are based on a single electron transfer reaction [27], their characteristics, sensitivities, mechanisms of the reaction, and endpoints are totally different. For instance, the DPPH method is based on the free radical scavenging activity, while FRAP measures the capability of reducing Fe^3+^ to Fe^2+^. Depending on what specific phytochemical constituents present in the extract are providing the antioxidant activity, their discrete chemical structures, positions, numbers, and types of substitutions can influence their redox properties and hence their antioxidant potentials [28].

Though both DPPH and FRAP assays are frequently used for assessing antioxidant capacity, they have some drawbacks. In the DPPH assay, interfering compounds may have significant absorption at the same measured wavelength. In addition, the DPPH radical is not present in living organisms. For the FRAP method, compounds with low redox potential, which probably do not serve as antioxidants* in vivo*, still can reduce the Fe^3+^. Interfering compounds may also absorb at the same wavelength, and the assay is also performed at a nonphysiological pH [29]. Therefore, antioxidant activities of EEP and WEP were also evaluated by the cell-based assay using an intracellular fluorescent probe, DCFH-DA. When the nonfluorescent DCFH-DA is taken up into cells, its diacetate moiety will be hydrolyzed by cellular esterases to generate the more polar DCFH which is trapped inside the cells. In the presence of ROS, intracellular DCFH is further oxidized to form the fluorescent DCF product [30]. The macrophage cell line RAW264.7 is usually the cell of choice in studying ROS-mediated cellular events since it can generate high amounts of ROS following an oxidant challenge. Catechin, resveratrol, and quercetin, at the level of concentration used in this study, have been shown and optimized to exhibit a strong suppression of intracellular ROS generation [22, 31, 32]. Therefore, the present study selected these compounds as antioxidant positive controls for the DCFH-DA assay. The present study demonstrated that all antioxidant standards, 250 *μ*M catechin, 20 *μ*M resveratrol, and 10 *μ*M quercetin, exerted a strong inhibition of ROS generation induced by tBuOOH over a period of 30 to 240 min. In addition to extracellular antioxidant capacity, EEP and WEP also served as intracellular ROS scavengers and subsequently decreased the oxidation of DCFH (Figures 3(b) and 3(c)). Both EEP and WEP were as efficient as the antioxidant standards in scavenging ROS. Notably, EEP was a better reducer of DCF fluorescence than WEP. The reduction of DCF fluorescence by EEP and WEP is not due to direct cytotoxicity as the range of concentration used in the studies had no effect on RAW264.7 cell viability (Figure 2).

Although the current study shows that an ethanol extract from PP leaves has* in vivo* anti-inflammatory activities [13], its mechanism of anti-inflammation is still unrevealed. Inflammatory disorders are characterized, among other events, by the production of significant amounts of free radicals, nitrogen reactive species, and pro-inflammatory cytokines [10]. High NO concentration combines with superoxides to form peroxynitrite ions (OONO^−^) which are responsible for cell and tissue damage from inflammation [33]. Therefore, we investigated inflammatory effects of EEP and WEP on the suppression of NO production in LPS plus IFN-*γ*-activated RAW264.7 cells. At the concentration range of 50–250 *μ*g/mL, both EEP and WEP dose-dependently suppressed NO production, and the suppression was more pronounced in EEP than WEP (Figure 4). These results agreed with the observation that EEP was also a better scavenger of intracellular ROS than WEP (Figures 3(b) and 3(c)).

As enhanced NO production by LPS and IFN-*γ*-stimulated RAW264.7 cells mainly occurs via increased intracellular content of iNOS [3, 4], the effect of EEP and WEP on iNOS expression was investigated. The present study clearly indicates that the suppressive effect of EEP and WEP on NO production was mediated through the inhibition of iNOS expression (Figures 5(a) and 5(b)). In agreement with the study of NO suppression, the suppressive effect of EEP (50–200 *μ*g/mL) on iNOS was more remarkable than that of WEP.

In addition to iNOS induction, LPS and IFN-*γ* also efficiently enhance COX-2 expression in RAW264.7 cells [3, 5]. An increased level of COX-2 expression is also known to account for the excessive production of PGE_2_ in most, if not all, inflammatory cells and tissues [34]. This study shows that both EEP and WEP can exhibit anti-inflammatory activity by reducing high COX-2 protein levels in a dose-related manner (Figures 5(c) and 5(d)). Thus EEP and WEP might play important roles in attenuating inflammation and cellular damage through their extra- and intracellular ROS scavenging activity and downregulation of NO, iNOS, and COX-2. Concordantly, Khumpook et al. [13] recently reported the* in vivo* anti-inflammatory activity of PP leaves as evidenced by decreased lipid peroxidation and NO level in concomitance with increased superoxide dismutase in the cotton-induced chronic inflammation in Albino rats, upon exposure to an ethanol extract of PP leaves for 17 days.

In fact, several medicinal plant extracts with natural antioxidant properties together with suppressive effects on NO, iNOS, and/or COX-2 expression in RAW264.7 have been reported to display a wide spectrum of bioactivities. These activities include anti-inflammation, such as curcumin from* Curcuma longa*, resveratrol from grape skins, red wines, and other plants, and a mixture of *β*-sitosterol and stigmasterol from* Andrographis paniculata* [8, 35, 36]. Previous investigators demonstrated that pretreatment of RAW264.7 with flavonoids such as apigenin, genistein, and kaempferol suppressed LPS-stimulated expression of NO, iNOS, and COX-2 protein production [37]. Major chemical constituents of PP leaves consist of *β*-sitosterol, stigmasterol, kaempferol 3-methyl ether 7-O-*β*-glucoside, and apigenin 7-O-*β*-glucoside [11]. All aforementioned compounds have been shown to possess anti-inflammatory properties. Both kaempferol 3-methyl ether 7-O-*β*-glucoside and apigenin 7-O-*β*-glucoside may be metabolized into kaempferol and apigenin which also have antioxidant and anti-inflammatory activities. Thus, it is possible that phenolic and flavonoid compounds in both EEP and WEP provide substantial antioxidant and anti-inflammatory activities.

In summary, the cytoprotective effects of EEP and WEP is due to their abilities to decrease ROS generation and NO radical production in cells. In addition, both EEP and WEP exert anti-inflammatory effects through the suppression of NO release and decrease the protein expression of iNOS and COX-2. Thus, PP leaves possess high potential for further exploration in the research development of anti-inflammatory medicine.

## Figures and Tables

**Table 1:** The percentage of recovery of crude extracts from fresh leaves of PP.

Extracts	Amount and source of preparation	Yield (g)	Percentage of recovery
EEP	1,500 g of fresh leaves	60.41	4.03 (from fresh leaves)
WEP	40 g of EEP	32.71	81.77 (from EEP)
	(993.21 g of fresh leaves)		3.29 (from fresh leaves)

**Table 2:** Total phenolic and flavonoid contents and total antioxidant (FRAP) activity of EEP and WEP.

Extracts	TPC	TFC	FRAP values			
	(mg GAE/g)	(mg CAE/g)	(mmol Fe^2+^/g)	(mg VCE/g)	(mg TRE/g)	(mg CAE/g)
EEP	200.14 ± 0.77^a^	109.67 ± 0.35^a^	2.87 ± 0.01^a^	213.23 ± 1.09^a^	292.54 ± 1.53^a^	133.25 ± 0.67^a^
WEP	212.47 ± 0.52^b^	118.06 ± 0.36^b^	2.61 ± 0.04^b^	193.40 ± 2.65^b^	264.70 ± 3.71^b^	121.05 ± 1.63^b^

Values are mean ± SEM (*n* = 3) and are representative of three independent experiments with similar results. Different letters within the same column are significantly different at *P* < 0.05 as determined by a Student''s *t*-test.
